# *Cirsiumtaiwanense* (Compositae, Cirsiumsect.Onotrophe, subsect. Australicirsium), a new species from Taiwan

**DOI:** 10.3897/phytokeys.183.70119

**Published:** 2021-10-12

**Authors:** Chih-Yi Chang, Hsy-Yu Tzeng, Yen-Hsueh Tseng

**Affiliations:** 1 Department of Forestry, National Chung-Hsing University, No. 145, Hsing-Ta Rd., Taichung 402, Taiwan National Chung-Hsing University Taichung Taiwan; 2 Taiwan Forestry Research Institute, No. 53, Nanhai Rd., Zhongzheng Dist., Taipei City, 10066, Taiwan Taiwan Forestry Research Institute Taipei Taiwan

**Keywords:** *Australicirsium* Kitam., central-northern Taiwan, *Cirsium*, karyotype analysis, pollen morphology, subsect

## Abstract

A new species of *Cirsium*, *C.taiwanense* Y.H.Tseng & Chih Y.Chang from central-northern Taiwan is reported in this article. This species is similar to *C.hosokawae* Kitam. in having a densely cobwebby abaxial leaf surface, but differs in its yellow (vs. vivid purplish red) corolla and the angle between the midrib and the lateral veins of the leaf, which is acute as opposed to nearly at a right angle in *C.hosokawae*. *Cirsiumtaiwanense* has 2*n* = 32 chromosomes, which is different from the other species in the Taiwanese subsect. Australicirsium Kitam. (2*n* = 34). An identification key to the *Cirsium* taxa of Taiwan is presented.

## Introduction

The genus *Cirsium* Mill. (Compositae) contains ca. 250 extant species, with its center of diversity in southern Europe and Caucasia (Werner 1976; Garcia-Jacas et al. 2002). This genus contains perennial, biennial, and annual spiny herbs, and has capitula with only disc florets, involucres of 5 to 20 series, setiferous receptacles, anther bases with caudate extensions, an achene apex with a short beak, and plumose pappus (Keil 2006; Funk et al. 2009). In East Asia, *Cirsium* has been reported in China (46 species, Shih and Greuter 2011), Japan (64 species, Iwatsuki et al. 1995), and Korea (8 species, Song and Kim 2007). According to Peng et al. (1998), nine species are recorded in Taiwan, one of which is represented by two varieties. Recently, Chang et al. (2019) described a new species endemic to Taiwan, namely *C.tatakaense* Y.H.Tseng & Chih Y.Chang, and Chang and Tseng (2019) reported a newly recorded variety, C.japonicum DC. var. fukienense Kitam. In addition, Chang and Tseng (2020) reported a newly naturalized species, *C.vulgare* (Savi) Tenore. The Taiwanese species are distributed from seashores to high altitude areas.

The island of Taiwan is located about 150 km off the southeast coast of China, between 21°45'N and 25°37'N, and 119°18'E and 122°06'E. Its climate ranges from tropical to subtropical. Taiwan is at the border between the paleotropical floristic kingdom and the Holarctic floristic kingdom (Good 1964; Takhtajan 1986). Several peaks exceed 3500 m a.s.l. and the highest is Mt. Yushan at 3952 m a.s.l., making Taiwan the fourth-highest island in the world (Chen 1980; Wang et al. 2009). Taiwan contains a diverse range of ecological niches in its mountains, which might have accelerated speciation and facilitated the evolution of endemic high-altitude plant species (Hsieh 2002). For example, there are seven native *Cirsium* species distributed from mid- to high altitudes, and all of them are endemic (Peng et al. 1998; Chang and Tseng 2019).

According to the infrageneric classification of East Asian *Cirsium* (Kitamura 1937; Shih 1984; Iwatsuki et al. 1995), the ten native species of Taiwan belong to three sections, viz. sect. Onotrophe (Cass.) DC., sect. Pseudoeriolepis (Nakai) Kitam., and sect. Spanioptilon (Less.) Shih. Section Onotrophe (Cass.) DC. is further subdivided into four subsections (Kitamura 1937), viz. subsect. Arenicola Kitam., subsect. Australicirsium Kitam., subsect. Nipponocirsium Kitam., and subsect. Sinocirsium Kitam. Following this infrageneric system, subsect. Australicirsium has only two species in Taiwan, *C.arisanense* Kitam. and *C.hosokawae* Kitam., which are characterized by erect or nodding capitula, phyllaries with a distinct midrib, and corolla lobes that are as long as the inflated part of the corolla tube (Kitamura 1937).

Recently, we discovered an unknown Cirsium belonging to subsect. Australicirsium (sect. Onotrophe) growing in the high mountain areas of central-northern Taiwan. This taxon appeared to be similar to *C.hosokawae*, with abaxial leaf surfaces covered with dense cobwebby hairs and by having nodding mature capitula. However, it can readily be distinguished from the latter by its yellow flowers, and the smaller angle between the midrib and the lateral veins. The aim of the present study was to elucidate the taxonomic status of this taxon using morphological, palynological and cytological approaches. After detailed examinations, we concluded that the taxon represents a new species and it is here described as *Cirsiumtaiwanense*.

## Materials and methods

### Morphological comparison

We compared the two Taiwanese taxa of subsect. Australicirsium with the unknown taxon. Morphological measurements were made using both fresh and dried specimens. For the morphological description, the terminology used by Peng et al. (1998) and Funk et al. (2009) was applied.

### Herbarium resources

Herbarium acronyms follow Index Herbariorum (Thiers 2021, continuously updated). Voucher specimens collected for the current study were deposited in PPI, TCF, and TNM. Specimens of the following herbaria were also examined: CHIA, HAST, KYO, PPI, TAI, TAIF, TCF, TI, TNM and TNU. The holotypes of both *C.arisanense* and *C.hosokawae* were also studied.

### Pollen morphology

Pollen grains were collected from fresh materials, and directly mounted on a stub. After air drying for 24 h at room temperature, the samples were sputter-coated with gold at 10–15 mA for 100 s (Quorum SC7620), and observed with a scanning electron microscope (Hitachi S-3400N). The shape, size and exine ornamentation were recorded using the methods of Erdtman (1952) and Halbritter et al. (2018). Information about voucher specimens is provided in Table 1.

**Table 1.** Voucher material for Cirsium Mill. subsect. Australicirsium Kitam. pollen morphology and karyotype analysis.

Taxa	Location	Altitude	Coordinate (WGS84)	Collecting date	Voucher number	Pollen morphology	Karyotype analysis
*C.arisanense*	Taiwan. Hualien County, Xiulin Township, Hsiaofengkou	2,996 m	24.16245°N, 121.28716°E	26 June 2015	*C. Y. Chang 602* (TNM)	●	●
	Taiwan. Taichung City, Heping District, Mt. Syue trail 8.9 km	3,399 m	24.39229°N, 121.24166°E	3 Sept. 2015	*C. Y. Chang 756* (TCF)	●	
	Taiwan. Nantou County, Ren’ai Township, Rueiyan river pipes road 2 km	2,215 m	24.11398°N, 121.20746°E	27 May 2016	*C. Y. Chang 1275* (TCF)	●	
	Taiwan. Nantou County, Sinyi Township, Tataka	2,609 m	23.47692°N, 120.89841°E	9 Feb. 2020	*C. Y. Chang 2733* (TCF)		●
*C.hosokawae*	Taiwan. Taichung City Heping District, Mt. Syue trail, Kupo to Mt. Syue East Peak	3,168 m	24.38882°N, 121.27348°E	8 Nov. 2015	*C. Y. Chang 870* (TNM)	●	
	Taiwan. Hualien County, Xiulin Township, Shangyuankuti, Mt. Nanhutashan	3,586 m	24.36042°N, 121.43713°E	5 Sept. 2016	*C. Y. Chang 1432* (TCF)	●	
	Taiwan. Ilan County, Datong Township, Mt. Nanhutashan trail, near Mt. Tochiatun	2,761 m	24.36801°N, 121.37971°E	11 Aug. 2018	*C. Y. Chang 2023* (TCF)		●
	Taiwan. Taichung City, Heping District, Mt. Nanhutashan trail, Sungfengling	2,648 m	24.36973°N, 121.37167°E	27 July 2019	*C. Y. Chang 2477* (TCF)	●	
	Taiwan. Taichung City, Heping District, Mt. Nanhutashan trail 8.5 km	2,707 m	24.36850°N, 121.37350°E	31 July 2019	*C. Y. Chang 2499* (TCF)	●	
*C.taiwanense*	Taiwan. Taichung City, Heping District, Mt. Syue trail 0.9 km	2,398 m	24.38520°N, 121.29254°E	3 July 2015	*C. Y. Chang 620* (TCF)	●	
	Taiwan. Nantou County, Ren’ai Township, Provincial Rd. No. 14A 21.5 km	2,657 m	24.11381°N, 121.22401°E	2 Oct. 2015	*C. Y. Chang 772* (TNM)	●	
	Taiwan. Taichung City, Heping District, Mt. Syue trail 0–1 km	2,353 m	24.38486°N, 121.29519°E	24 Apr. 2016	*C. Y. Chang 1230* (TCF)		●
	Taiwan. Taichung City, Heping District, Mt. Tao	2,648 m	24.41766°N, 121.30693°E	22 July 2018	*C. Y. Chang 1926, 2133* (TCF)		●

### Karyotype analysis

Karyotype analysis was performed using the procedures of Ozcan et al. (2011) and Yüksel et al. (2013). Root tips were collected on sunny mornings and pre-treated with 2 mM 8-hydroxyquinoline below 4 °C for 8 h, then fixed with Carnoy’s solution (absolute ethanol:acetic acid, 3:1, v:v) for at least 24 h at 0 °C. The fixed roots were then stained with 2% aceto-orcein for 24 h at room temperature, squashed, and the slides were examined using an optical microscope (Accu-Scope 3025) equipped with a CCD camera (ProgRes C14 plus). Information about voucher materials is presented in Table 1.

### Distribution map

A distribution map was generated using QGIS ver. 3.4 from the package developed by Lin (2018). Geographical climatic regions and altitudinal vegetation zones of Taiwan were indicated following Su (1984, 1985) (Fig. 4) . The geographical range of each species was determined from information on herbarium specimens.

### Data analysis

The values of the quantitative morphological and palynological traits were determined and their means and standard deviations were calculated (Table 2). Differences between taxa were analyzed using a one-way ANOVA, followed by Tukey’s HSD multiple-range test (*p* ≤ 0.05) (Zar 1984). All analyses were performed using the PASW Statistics ver. 18 software (Sarma and Vardhan 2018).

**Table 2.** Summary of diagnostic characters of Cirsium Mill. subsect. Australicirsium Kitam. in Taiwan.

Characters		*C.arisanense*	*C.hosokawae*	*C.taiwanense*
Rosette leaves	Size (cm)	31.32 ± 13.71^a^ × 5.55 ± 1.27^a^	19.69 ± 3.79^b^ × 4.42 ± 1.02^a^	26.05 ± 6.18^ab^ × 5.55 ± 1.27^a^
	Shape	narrowly elliptic	narrowly elliptic to oblanceolate	narrowly elliptic to oblanceolate
	Angle between midrib and lateral vein (°)	68.78 ± 5.12^b^	83.05 ± 9.88^a^	63.04 ± 12.00^b^
	Cobwebby hairs on abaxial leaf surface	Absent	present	present
Cauline leaves	Size (cm)	12.70 ± 5.84^b^ × 4.10 ± 2.95^a^	17.88 ± 0.78^a^ × 5.26 ± 0.74^a^	16.17 ± 5.01^ab^ × 4.85 ± 1.56^a^
	Angle between midrib and lateral vein (°)	65.31 ± 9.06^b^	83.73 ± 9.53^a^	62.30 ± 10.71^b^
	Cobwebby hairs on abaxial leaf surface	Absent	present	present
Capitula	Mature capitula	erect or sometimes nodding	nodding	nodding
	Size (cm)	3.05 ± 0.22^a^ × 1.42 ± 0.25^ab^	2.60 ± 0.66^a^ × 1.18 ± 0.32^b^	3.03 ± 0.22^a^ × 1.61 ± 0.17^a^
Phyllaries	Length ratio (inner vs. outer)	2.05 ± 0.75^ab^	1.46 ± 0.33^b^	2.91 ± 0.73^a^
	Length of the reflexed part of the phyllaries (mm)	4.88 ± 1.23^a^	2.33 ± 0.80^b^	2.30 ± 0.18^b^
	Number	81.3 ± 11.5^b^	86.3 ± 12.0^b^	111.7 ± 13.3^a^
Florets	Length (cm)	2.54 ± 0.15^ab^	2.46 ± 0.21^b^	2.63 ± 0.21^a^
	Inflated part of corolla tube length (mm)	4.39 ± 0.63^b^	4.41 ± 0.46^b^	5.11 ± 0.70^a^
	Corolla color	Yellow	vivid purplish red	yellow
	Corolla lobes	Revolute	erect	erect
	Anther length (mm)	6.02 ± 0.78^b^	6.10 ± 0.51^b^	6.86 ± 0.80^a^
	Number	102.7 ± 24.1^ab^	84.6 ± 19.5^b^	129.7 ± 30.1^b^
Achene	Size (mm)	3.77 ± 0.16^a^ × 1.49 ± 0.06^ab^	3.95 ± 0.13^a^ × 1.37 ± 0.05^b^	3.97 ± 0.18^a^ × 1.65 ± 0.07^a^
	Pappus length (cm)	1.66 ± 0.04^a^	1.29 ± 0.04^b^	1.60 ± 0.05^a^
Pollen	Pollen size (P/E, μm)	47.61 ± 0.80^a^ / 46.37 ± 1.00^a^	41.40 ± 0.60^b^ / 41.15 ± 0.75^b^	48.50 ± 0.80^a^ / 47.00 ± 1.00^a^
	Pollen spine length (μm)	4.25 ± 0.18^a^	3.91 ± 0.14^ab^	3.20 ± 0.18^bc^
	Pollen spine base width (μm)	4.44 ± 0.26^a^	5.33 ± 0.19^a^	4.74 ± 0.26^a^
Chromosome number		2*n* = 34	2*n* = 34	2*n* = 32
Distribution		Endemic to Taiwan; widely distributed in open mountain areas at 1500–3800 m a.s.l. (Chang et al. 2019).	Endemic to Taiwan; open areas at 1400–3600 m a.s.l. in central-northern Taiwan.	Endemic to Taiwan; open areas at 1400–3400 m a.s.l. in central-northern Taiwan.

^abc^ Means in the same row followed by the same letter are not significantly different (*p* ≤ 0.05; Tukey’s HSD test).

## Results

### Macro-morphological differences

**Figure 1.**
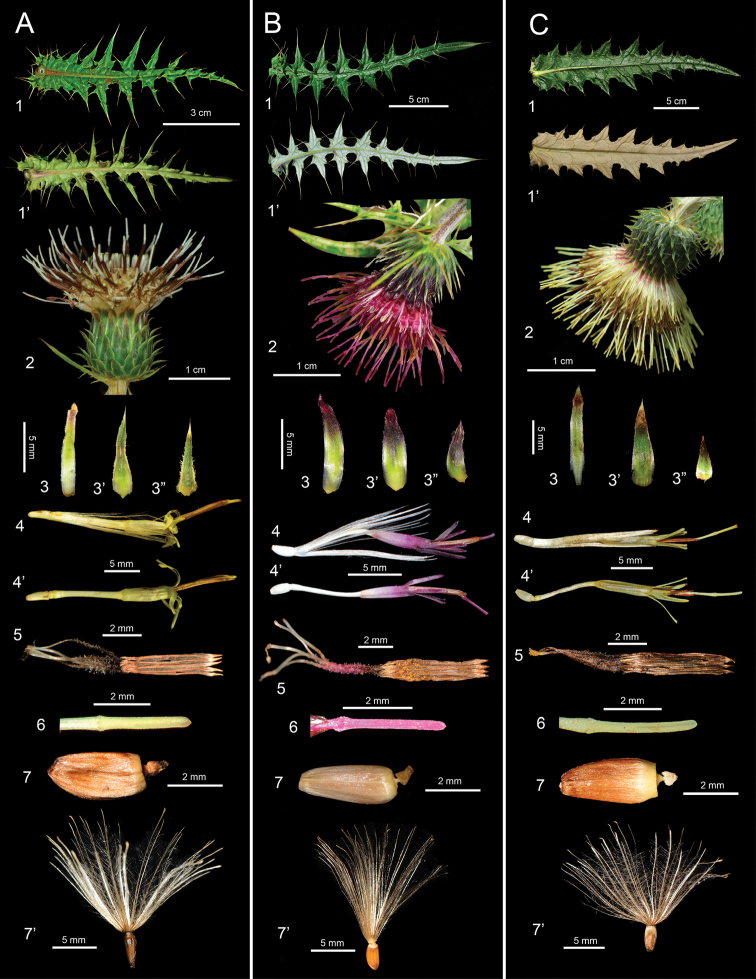
Comparison of the morphological characters of Cirsium Mill. subsect. Australicirsium Kitam. **A***C.arisanense* Kitam **B***C.hosokawae* Kitam **C***C.taiwanense* Y.H.Tseng & Chih Y.Chang **1** cauline leaf, adaxial view **1**' cauline leaf, abaxial view **2** capitulum **3** inner phyllary **3**' middle phyllary **3**" outer phyllary **4** floret **4**' floret (pappus removed) **5** synantherous stamens **6** style branches **7** achene **7**' achene with pappus.

The abaxial leaf surface of the members of subsect. Australicirsium in Taiwan displays two types of indumentum. Both *C.hosokawae* and *C.taiwanense* are densely covered with cobwebby hairs, whereas *C.arisanense* is without cobwebby indumentum. The angle between the midrib and the lateral veins of the leaves of *C.hosokawae* is often almost 90°, (60–)82–90°, which differs significantly (*p* ≤ 0.05) from that of *C.arisanense*, (49–)57–78° and *C.taiwanense* (44–)52–73° (Fig. 1, Table 2). In addition, the mature capitula of *C.arisanense* are erect and rarely nodding, whereas those of *C.hosokawae* and *C.taiwanense* are usually nodding. *Cirsiumtaiwanense* has significantly (*p* ≤ 0.05) more florets in a capitulum (101–135(–194)) than *C.arisanense* (78–137) and *C.hosokawae* (54–111), and a larger number of phyllaries: 90–127 vs. 66–100 for *C.arisanense* and 68–109 for *C.hosokawae*. (Fig. 1, Table 2). Further, the corolla of *C.taiwanense* and *C.arisanense* is yellow, but that of *C.hosokawae* is vivid purplish red. Although the color of the corolla of *C.hosokawae* could not be determined from its type specimen, it is described as red in the protologue (Kitamura 1932). The populations described here have yellow corollas and are therefore regarded as *C.taiwanense*. Moreover, the corolla lobes of *C.arisanense* are revolute, whereas the two other species have erect corolla lobes (Fig. 1, Table 2). Finally, the pappus of the achene of *C.hosokawae* is significantly (*p* ≤ 0.05) shorter (1.02–1.48 cm) than that of *C.arisanense* (1.44–1.73 cm) and *C.taiwanense* (1.55–1.66 cm) (Fig. 1, Table 2). In general, the leaves of *C.taiwanense* and *C.hosokawae* are similar, as the abaxial leaf surfaces of both species are covered with dense cobwebby hairs. Therefore, herbarium specimens are often misidentified. Our field observations however suggest that the color of the corolla and the angle between the midrib and the lateral veins of the leaves are reliable characters for distinguishing the two species.

### Pollen morphology

**Figure 2.**
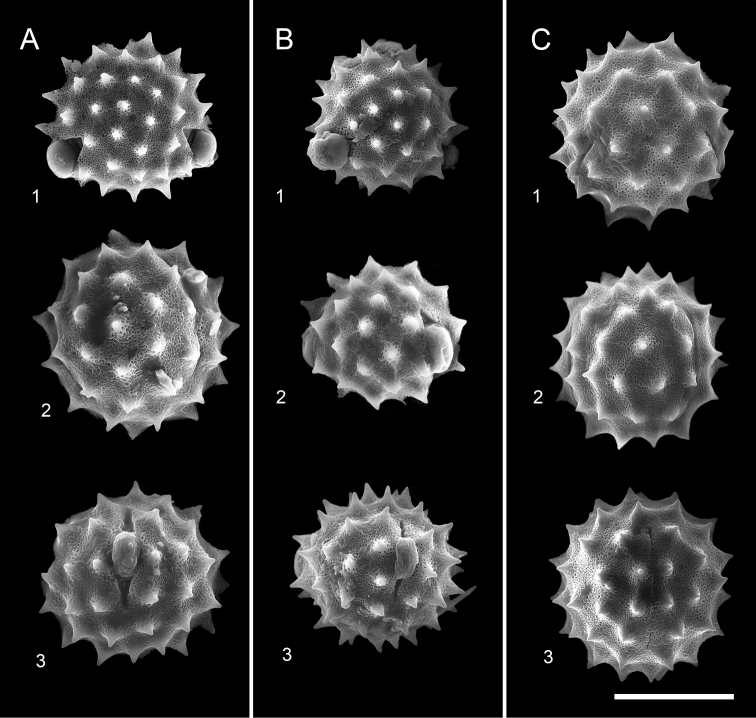
Comparison of the pollen morphology of Cirsium Mill. subsect. Australicirsium Kitam **A***C.arisanense* Kitam **B***C.hosokawae* Kitam **C***C.taiwanense* Y.H.Tseng & Chih Y.Chang **1** polar view **2** equatorial view **3** colporate view. Scale bar: 30 μm.

The pollen grains of Taiwanese species of subsect. Australicirsium are tricolporate, spheroidal and of medium size. The pollen grains of *C.taiwanense* (43.5–51.8 μm) and *C.arisanense* (42.9–53.0 μm) have a significantly (*p* ≤ 0.05) larger diameter than those of *C.hosokawae* (36–46 μm) (Fig. 2B). *Cirsiumarisanense* has significantly (*p* ≤ 0.05) longer pollen spines (2.9–5.1 μm) (Fig. 2A) than *C.taiwanense* (2.8–3.6 μm), but these are not significantly (*p* ≤ 0.05) longer than those of *C.hosokawae* (3.2–4.9 μm) (Fig. 2C, Table 2).

### Chromosome cytology

**Figure 3.**
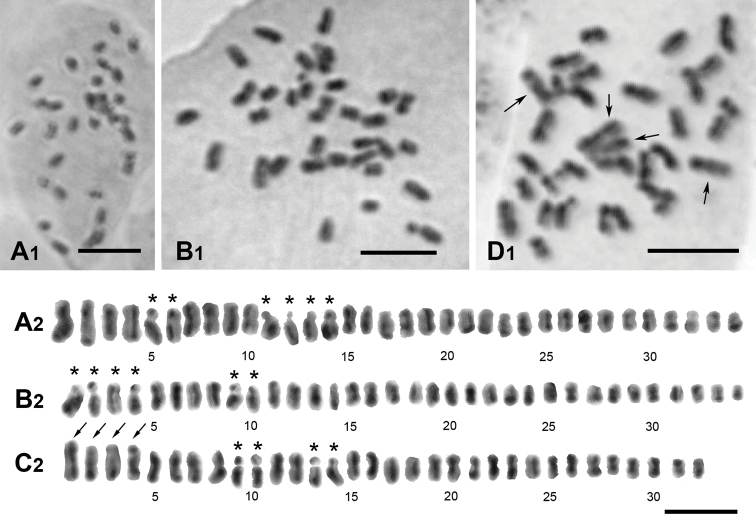
Karyotype of Cirsium Mill. subsect. Australicirsium Kitam **A***C.arisanense* Kitam., 2*n* = 34 **B***C.hosokawae* Kitam., 2*n* = 34 **C***C.taiwanense* Y.H.Tseng & Chih Y.Chang, 2*n* = 32 **1** cell **2** karyogram. *relatively clear satellites; arrow indicates chromosomes with secondary constriction. Scale bar: 5 μm.

The most common chromosome number of *Cirsium* species is 2*n* = 34 (Hsu 1970; Funk et al. 2009; Chen and Yeh 2010a, 2010b). Our cytological investigation also shows that the chromosome numbers of both *C.arisanense* and *C.hosokawae* are 2*n* = 34 (Fig. 3A, B). In contrast, the chromosome number of *C.taiwanense* is 2*n* = 32 (Fig. 3C).

Although the chromosomes of the three taxa were too short to determine their karyotypic formula, satellites and secondary constrictions could be observed in longer chromosomes. Satellites were observed in *C.arisanense* for the 3^rd^, 6^th^ and 7^th^ pairs (Fig. 3A), in *C.hosokawae* for the 1^st^, 2^nd^ and 5^th^ pairs (Fig. 3B), and in *C.taiwanense* for the 5^th^ and 7^th^ pairs (Fig. 3C). In addition, only the 1^st^ and 2^nd^ pairs of *C.taiwanense* have secondary constrictions (arrows in Fig. 3C). Our results show that each taxon of subsect. Australicirsium in Taiwan has a clearly different karyotype (Fig. 3).

### Distribution

**Figure 4.**
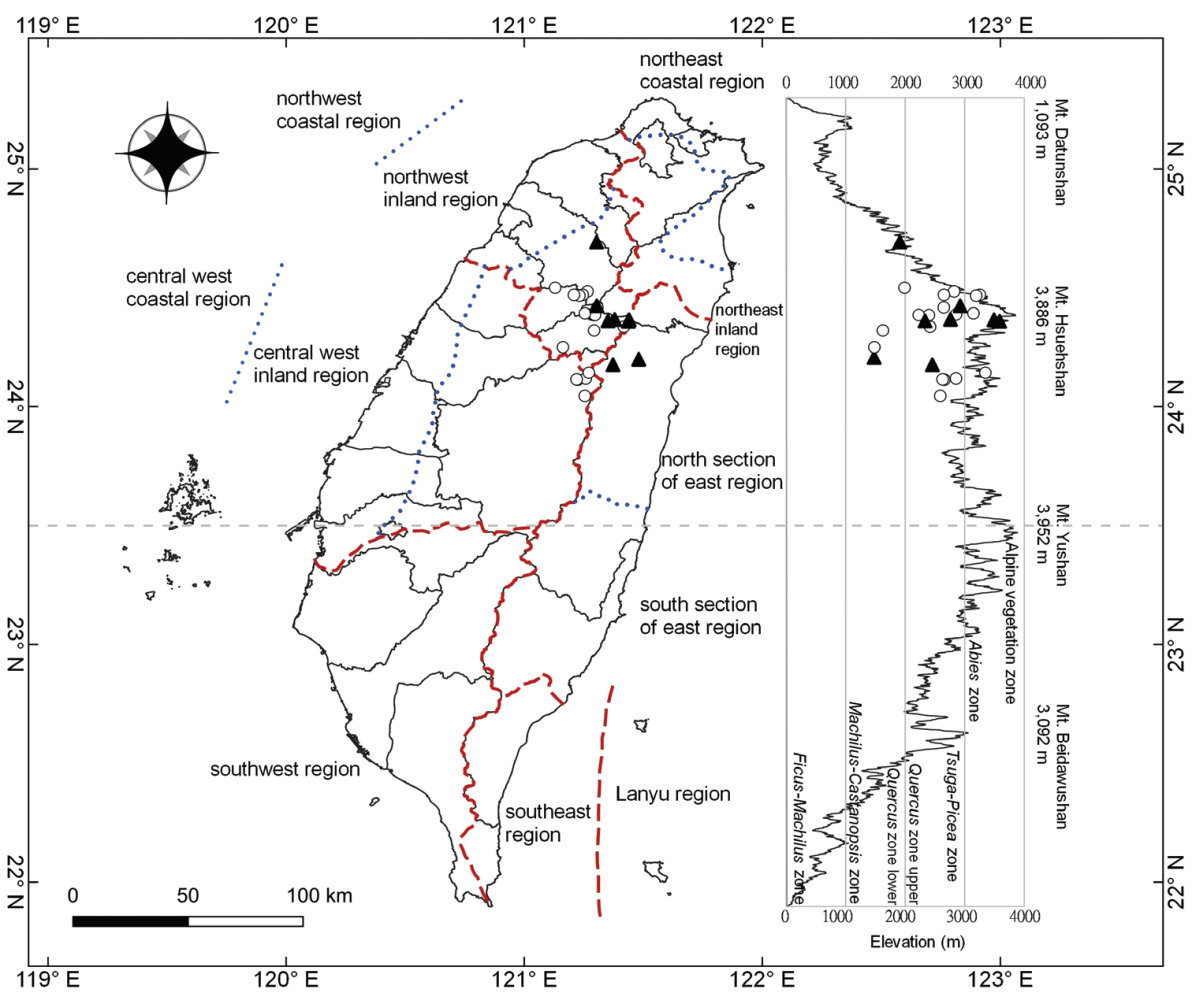
Distribution map of *Cirsiumhosokawae* Kitam. (▲) and *C.taiwanense* Y.H.Tseng & Chih Y.Chang (○) in Taiwan. The red interrupted lines indicate major geographical climate regions as per Su (1985), and the blue dotted lines indicate sections within each climate region. The right part of the figures shows a side view of Taiwan and the vertical lines indicate altitudinal vegetation zones as per Su (1984).

*Cirsiumarisanense* is the most common *Cirsium* species in high altitude regions in Taiwan (see distribution map in Chang et al. 2019). In comparison, *C.hosokawae* and *C.taiwanense* are less common and widespread. Both *C.hosokawae* and *C.taiwanense* occupy similar habitats and altitudes, often occurring in open areas such as in wide roadsides and forest margins at 1400–3600 m a.s.l. However, the latitudinal distributions of the two species are different. *Cirsiumhosokawae* and *C.taiwanense* are mainly found in the northwest inland region (Su 1985). However, *C.hosokawae* is absent from the central west inland region, whereas *C.taiwanense* is found less frequently near the western boundary of the north section of the east region. In general, the distribution of *C.taiwanense* is concentrated in the southwest and *C.hosokawae* is in the northeast of their combined distribution area (Fig. 4). The climate of the *C.hosokawae* habitat is usually more humid than that of *C.taiwanense*.

## Discussion

### The differences and the taxonomic status of the unknown *Cirsium*

*Cirsiumtaiwanense* has a unique combination of morphological characteristics: its corolla lobes are erect and yellow, and the abaxial surface of the leaves is densely covered with cobwebby hairs (Fig. 1C). Additionally, *C.taiwanense* has the largest pollen grains and shortest pollen spines of the three species of subsect. Australicirsium in Taiwan (Fig. 2, Table 2). The chromosome number of *C.taiwanense* is 2*n* = 32 (Fig. 3C), which is different from the other known *Cirsium* species in Taiwan (Hsu 1970; Peng and Hsu 1978; Chen and Yeh 2010a, 2010b; Chang et al. 2019). Also, the 1^st^ and 2^nd^ pairs of chromosomes in its karyotype have secondary constrictions, which is different from other subsect. Australicirsium species (Fig. 3). Based on the above comparison, *C.taiwanense* is clearly different from other known congeners. We therefore here describe *C.taiwanense* as a new species.

### Key to the 14 wild *Cirsium* taxa of Taiwan (modified from Chang et al. (2019), Chang and Tseng (2019), and Chang and Tseng (2020)

**Table:** 

1	Biennial herb; leaves villose; involucre cylindrical or urceolate	**2**
2	Stem without wings; mature capitula nodding; involucre cylindrical	***C.ferum***
2*	Stem with spiny wings; mature capitula erect; involucre urceolate	***C.vulgare*^1^**
1*	Perennial herb; leaves glabrous, pubescent or densely cobwebby; involucre urceolate or cupuliform	**3**
3	All leaves cauline, basal rosette leaves absent	**4**
4	Leaves densely cobwebby on abaxial surface; mature capitula erect, involucre urceolate; apical parts of inner phyllaries inflated, obtuse; outer phyllaries lanceolate, apex acute without a spine; corolla lobes obviously longer than the inflated part of corolla tube	***C.lineare***
4*	Leaves glabrous on both surfaces; mature capitula nodding, involucre cupuliform; apical parts of inner phyllaries acute or acuminate; outer phyllaries elliptical with a long spine at the apex; corolla lobes as long as the inflated part of corolla tube	**5**
5	Corollas white; leaves pinnatisect or bipinnatisect, lobes > 1.5 cm wide	***C.kawakamii***
5*	Corollas purple; leaves mainly pinnatisect, lobes < 1.2 cm wide	***C.tatakaense***
3*	Leaves in both a basal rosette and cauline	**6**
6	Reflexed part of the phyllaries blade-like; corolla lobes as long as the inflated part of corolla tube	**7**
7	Corollas white or light purple	**8**
8	Corollas white; inner and outer phyllaries similar in length; stems cauline, without rhizome	***C.brevicaule***
8*	Corollas light purple; inner and outer phyllaries distinctly different in length; stems both cauline and rhizomatous	***C.morii***
7*	Corollas yellow or vivid purplish red	**9**
9	Abaxial leaf surface without cobwebby indumentum; mature capitula erect or nodding; corolla lobes revolute	***C.arisanense***
9*	Abaxial leaf surface densely cobwebby; mature capitula nodding; corolla lobes erect	**10**
10	Corolla vivid purplish red; angle between midvein and lateral veins of leaf (60–)82–90°	***C.hosokawae***
10*	Corolla yellow; angle between midvein and lateral veins of leaf (44–)52–73°	***C.taiwanense***
6*	Reflexed part of the phyllaries spine-like; corolla lobes shorter than the inflated part of corolla tube	**11**
11	Abaxial leaf surface densely cobwebby; mature capitula nodding	***C.suzukii***
11*	Leaf surfaces pubescent, but not cobwebby; mature capitula erect	**12**
12	Corolla white; leaves glabrescent	**C.japonicumvar.takaoense**
12*	Corolla purple; leaves villose, not glabrescent	**13**
13	Apical spines of phyllaries shorter than 2 mm; leaves pinnatifid to pinnatisect	**C.japonicumvar.australe**
13*	Apical spines of phyllaries longer than 3 mm; leaves pinnatipartite to pinnatisect	**C.japonicumvar.fukienense**

### Taxonomic treatment

#### Cirsium taiwanense

Taxon classificationPlantaeAsteralesAsteraceae

Y.H.Tseng & Chih Y.Chang sp. nov.

10B2969B-F633-5D88-B90B-F88C9B6AA244

urn:lsid:ipni.org:names:77220552-1

**Figure 5.**
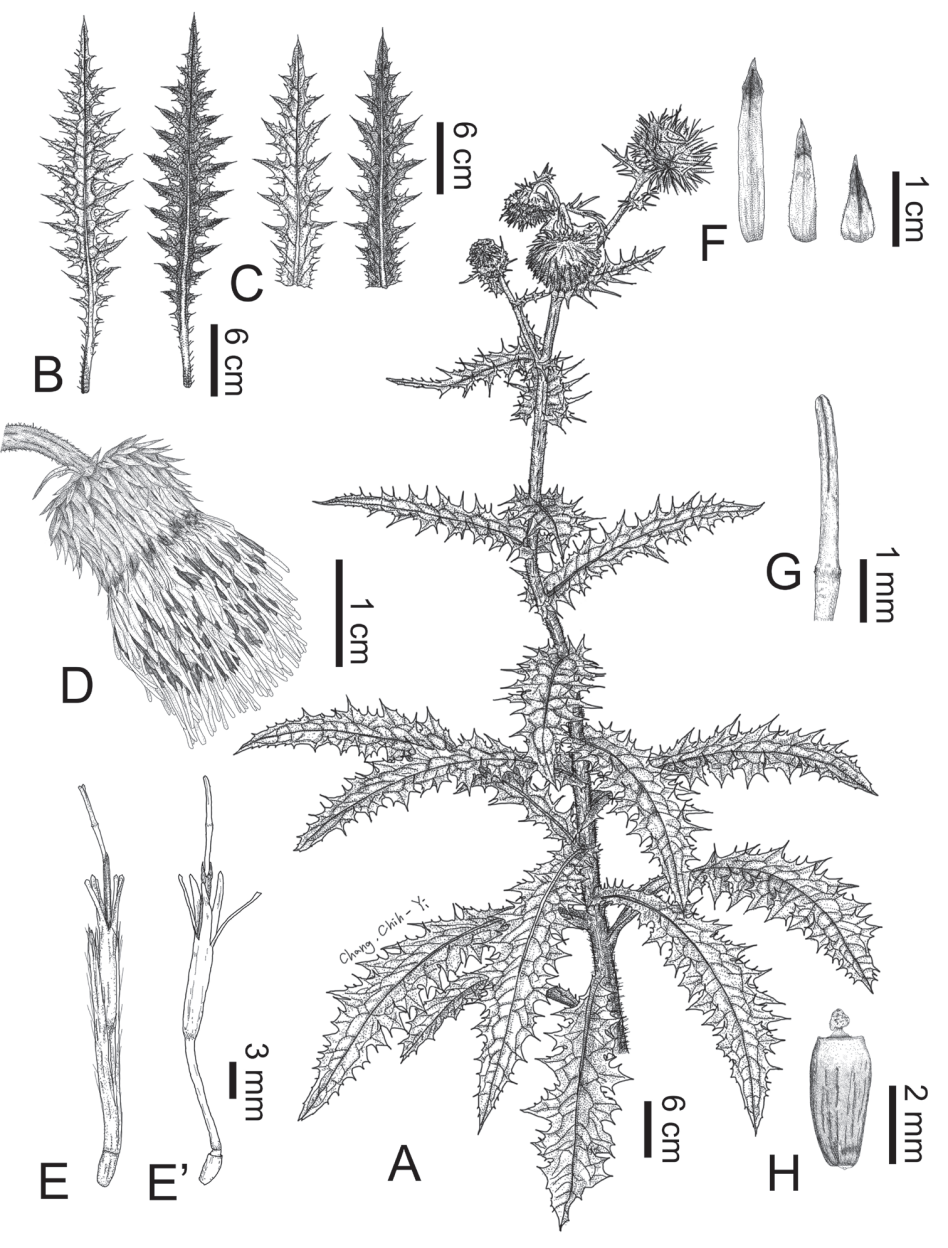
Line drawings of *Cirsiumtaiwanense* Y.H.Tseng & Chih Y.Chang **A** habit **B** rosette leaf **C** cauline leaf **D** capitulum **E** floret **E**’ floret (pappus removed) **F** phyllaries **G** style branches **H** achene.

**Figure 6.**
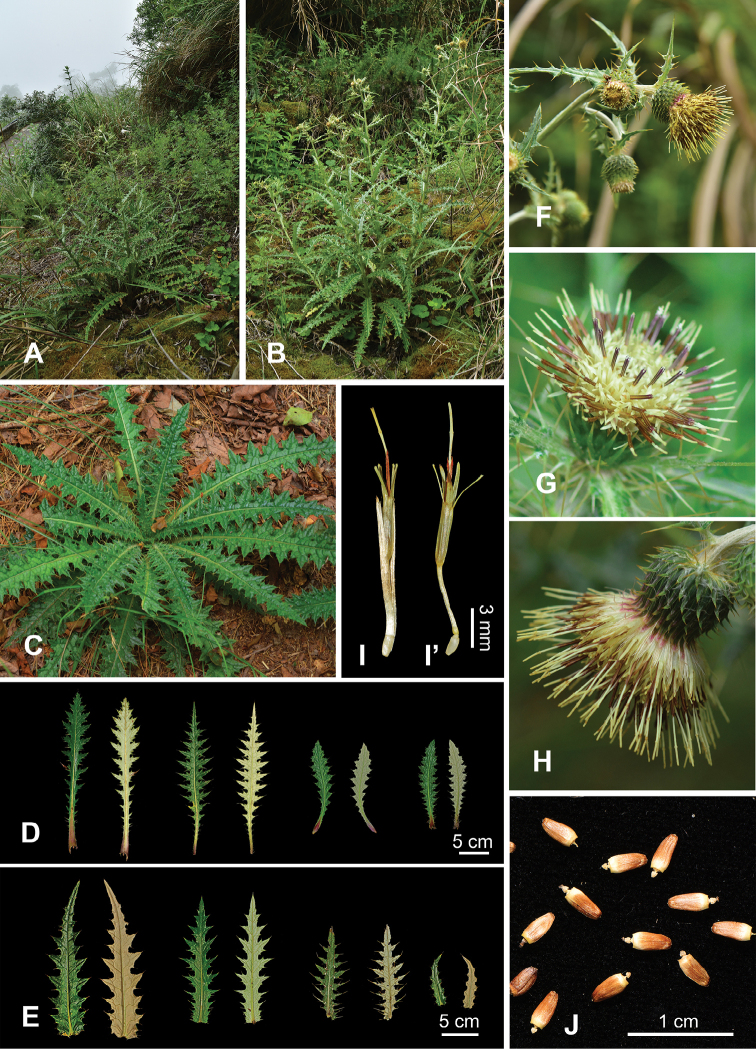
*Cirsiumtaiwanense* Y.H.Tseng & Chih Y.Chang **A** habitat **B** habit **C** basal rosette **D** variations of rosette leaves **E** variations of cauline leaves **F** inflorescences **G, H** capitulum **I** floret **I**’ floret (pappus removed) **J** achenes.

Figures 1C , 2C , 3C , 5 , 6

##### Diagnosis.

Differs from *C.hosokawae* in having a yellow corolla (vs. vivid purplish red corolla), a narrower angle between the midrib and lateral veins of the cauline leaves ((44–)52–73° vs. 82–90°), and usually more florets (101–135(–194) vs. 54–111) and phyllaries (90–127 vs. 68–109) per capitulum. Differs from *C.arisanense* by its nodding mature capitula (vs. erect), erect corolla lobes (vs. revolute), and a densely cobwebby abaxial leaf surface (vs. without cobwebby indumentum).

##### Type.

Taiwan. Nantou County, Ren’ai Township, Provincial Rd. No. 14A 21 km, 2605 m alt., 24.11438°N, 121.21821°E, 15 July 2020. *C. Y. Chang 2976* (holotype: TCF; isotype: TNM, PPI).

##### Description.

Perennial herbs, stems 0.5–1.0 m tall, internodes terete. Leaves pinnatipartite or pinnatisect, space between pinnae V-shaped, adaxial surface puberulent or cobwebby, abaxial surface densely cobwebby, margin spinose; rosette leaves narrowly elliptic to oblanceolate, base cuneate to attenuate, apex narrowly acute, 19.5–34.1 × 4.1–7.4 cm, angle between the midrib and the lateral veins (40–)55–76°; pinnae 7–11 pairs, 0.9–2.6 ×1.2–2.1 mm, space between pinnae 0.4–1.2 cm, petiole 1.5–4.0 cm; cauline leaves narrowly elliptic to narrowly triangular, base cordate, apex narrowly acute, 9.0–25.5 × 2.2–6.9 cm, angle between the midrib and the lateral veins (44–)52–73°; pinnae 5–8 pairs, 1.3–2.1 × 0.8–1.9 cm, space between pinnae 0.6–1.5 cm, sessile. Capitula solitary or 2–6 arranged into racemes or panicles, mature capitula nodding. Involucre urceolate, more or less cobwebby, 2.8–3.4 × 1.4–1.9 cm; phyllaries 90–127, in 5–7 series, apex acute, midrib distinct, (0.3–)0.5–1.4 × 1.5–2.1 mm, length ratio of inner and outer phyllaries 2.2–3.6, the reflexed part of the phyllaries 1.5–3.1 mm long. Receptacle flat, densely bristly. Florets 101–135(–194), 2.2–3.1 cm long, with yellow corolla, corolla lobes 5, linear, erect, 3.0–5.1 × 0.4–0.7 mm, corolla tube fistulose with 2 sections, the inflated section of corolla tube 4.1–5.8 mm long; synantherous stamens 5, anthers brown, 5.9–8.3 mm long, base with caudate extensions, filaments 3.0–4.5 mm long with irregular protuberances; stigmas bifid, styles 2.0–2.5 cm long, style arm 2.6–2.7 mm long, ovaries (1.6–)3.2–4.1 mm long. Achenes oblong, base acute, apex truncate, beige, 3.5–4.5 × 1.5–1.8 mm, compressed, 4-angled, ribbed, beak heart-shaped; pappus copious, plumose, bristles in many series, 1.55–1.65 cm long, forming basal ring, easily shed.

##### Phenology.

Flowering between June and October and fruiting between July and November.

##### Distribution and habitat.

Endemic species of Taiwan. *Cirsiumtaiwanense* grows in open areas between *Querus* forest and *Abies* forest at 1400–3400 m a.s.l. in central-northern Taiwan. *Cirsiumtaiwanense* usually grows at sunny sites. Common companion species are *Artemisiamorrisonensis* Hayata (Compositae), *Liliumformosanum* Wallace (Liliaceae), *Salixfulvopubescens* Hayata (Salicaceae) and *Rubuspectinellus* Maxim. (Rosaceae).

##### Chinese name.

Tai-wan-ji (臺灣薊).

##### Chromosome number.

2*n* = 32 (Fig. 3B).

##### Palynology.

Pollen grains are tricolporate, spheroidal, micro-reticulate and 46.6–51.8 × 43.5–50.7 μm (P/E ratio: 0.9–1.1). The surface is densely covered with spines that are 2.8–3.6 μm long and 3.9–6.0 μm wide at the base. The distance between spines is 8.2–11.8 μm (Fig. 2C).

##### Conservation status.

*Cirsiumtaiwanense* is common in north-central Taiwan (Fig. 4). The populations often grow in high mountain areas and experience limited disturbance by humans. Following the International Union for Conservation of Nature (IUCN) Categories and Criteria (IUCN 2019), we regard this species as of Least Concern (LC).

##### Additional specimens examined.

Taiwan. Hsingchu County, Jianshi Township, Mt. Itsashan, 7 Sept. 1993. *C. L. Huang 78* (HAST!). Miaoli County, Taian Township, en route from 99 lodge to Mt. Tapachienshan, 11 Aug. 1985. *C. I Peng 8492, 8542, 8543, 8544, 8545, 8546* (HAST!); same loc., 2 Nov. 1996. *C. M. Wang 2384* (TNM!); Taian, 13 Sept. 1996. *C. H. Chen 1870* (TAIE!); 99 Lodge, 2780 m alt., 2 Nov. 1996. *C. M. Wang 2384* (HAST!); Mt. Hsishihshan, 27 Oct. 1976. *B. P. Yang 81* (TAIF!). Taichung City, Heping District, Mt. Nanhutashan, 21 Sept. 1969. *T. Yamazaki 281* (TI!); Yunleng cabin to Mt. Duojiatunshan, 26 June 1994. *C. M. Wang 1026* (TNM!); en route from entrance to Yunleng cabin, 27 July 2019. *C. Y. Chang 2478* (TCF); Derji, 3 July 1974. *C. I Peng 15* (TAI!); en route from entrance to Chika Lodge, 1 June 2003. *C. M. Wang 6908* (TNM!); same loc., 19 June 2009. *C. M. Wang 13025* (TNM!); same loc., 20 Jun. 2011. *C. I Huang 5273* (HAST!); same loc., 16 July 2009. *Y. H. Tseng 4697* (TCF); same loc., 9 Feb. 2021. *C. Y. Chang 3269* (TCF); en route from Chika lodge to Mt. Syue east peak, 7 Aug. 1986. *C. I Peng 9660* (HAST!); same loc., 11 Sept. 2002. *C. I Huang 1238* (HAST!; TNM!); same loc., 21 June 2011. *C. I Huang 5290* (HAST!); en route from Chika lodge to 369 Lodge, 15 June 1985. *C. I Peng 7887* (HAST!); behind 369 Lodge, margin of *Abies* forest, 9 Sept. 2009. *C. T. Chao 922* (TCF!); en route from Wuling lodge to Mt. Tao, 24 Aug. 1988. *C. I Peng 12012, 12020, 12100* (HAST!); same loc., 22 July 2018. *C. Y. Chang 2133* (TCF!); en route from Wuling lodge to Taoshan waterfall, 24 Aug. 1988. *C. I Peng 12097* (HAST!); Huanshan, 6 July 2006. *Z. H. Chen 186* (TAIF!). Nantou County, Ren’ai Township, Guandaoxi, 22 Oct. 1932. *S. Sasao s. n.* (CHIA!); Hsinjenkang, Provincial Rd. No. 14A 21 km, 8 Sept. 1997. *S. H. Wu 384* (HAST!); Provincial Rd. No. 14A 22 km, 3 Aug. 2011. *T. W. Hsu 17059* (TAIE!); Mt. Hohuanshan near Yuanfeng, 13 July 1985. *C. I Peng 8327* (HAST!); Nenggao Cross-ridge Historic Trail, 28 Feb. 2006. *M. J. Chung x22805* (TAIF!); same loc., 31 Jan. 2018. *C. Y. Chang 1609* (TNM); Provincial Rd. No. 14A 21.5 km, 2 Oct. 2015. *C. Y. Chang 772* (TNM); same loc., 27 Oct. 2015. *C. Y. Chang 839* (TNM).

## Supplementary Material

XML Treatment for Cirsium taiwanense

## Footnotes

naturalized species.
